# Antibacterial and Antibiofilm Potency of XF Drugs, Impact of Photodynamic Activation and Synergy With Antibiotics

**DOI:** 10.3389/fcimb.2022.904465

**Published:** 2022-06-30

**Authors:** Emma Louise Board-Davies, William Rhys-Williams, Daniel Hynes, David Williams, Damian Joseph John Farnell, William Love

**Affiliations:** ^1^ School of Dentistry, Cardiff University, Cardiff, United Kingdom; ^2^ Destiny Pharma plc, Brighton, United Kingdom

**Keywords:** XF drugs, photodynamic therapy, antibacterial, antimicrobial resistance, biofilms

## Abstract

With increasing incidence of antimicrobial resistance, there is an urgent need for novel and effective antibacterials. Destiny Pharma plc have developed a series of porphyrin-based XF drugs, some with dual mechanisms of antibacterial action. An innate mechanism acts through binding to the outer bacterial membrane and a separate, light-activated, photodynamic (PD) mechanism, acts *via* the generation of reactive oxygen species. This study aimed to assess the innate and PD associated antibacterial activity of XF drugs against planktonic bacteria, their biofilms and combinational effects with conventional antibiotics. Minimum inhibitory concentrations (MICs) were determined for 3 XF drugs against 114 bacterial isolates. MICs for XF-73 and XF-70 were determined (± PD). DPD-207 was designed to not exhibit PD action due to its structure. XF-drugs (± PD) were further assessed for synergy with conventional antibiotics (using a checkerboard assay) and antibiofilm activity against susceptible strains. XF drugs were innately active against all tested Gram-positive isolates. PD action significantly increased bacterial susceptibility to XF-73 and XF-70 for all Gram-positive isolates. Generally, the XF drugs exhibited higher MICs against Gram-negative isolates, however PD significantly enhanced potency, particularly for XF-70. XF-73 and XF-70 exhibited synergy with ertapenem against a methicillin resistant *Staphylococcus aureus* (MRSA) strain (± PD) and XF-73 with polymyxin B (± PD) against *Pseudomonas aeruginosa*. No antagonism was seen between the XF drugs and any of the 5 antibiotics tested. The antibiofilm effect of XF drugs was also observed for all *Staphylococcus* isolates tested. Generally, PD did not enhance activity for other bacterial isolates tested with the exception of XF-73 against *Acinetobacter baumannii* biofilms. XF drugs exhibited significant antimicrobial activity against Gram-positive bacteria, with PD enhancement of bacterial susceptibility. Additionally, XF drugs displayed synergy with conventional antibiotics and demonstrated antibiofilm effects.

## Introduction

In the last century, antimicrobial therapy revolutionised the management of infectious diseases to such an extent that previously untreatable and life-threatening conditions became curable. However, after only 70 years of antibiotic use, the original perception that antimicrobial therapy would ‘end’ infectious diseases has not materialised. The failure of antimicrobial therapy as the ‘panacea’ of human infection, has primarily been due to antimicrobial resistance (AMR), first seen with resistance to penicillin after only one year following its introduction. (Rammelkamp and Maxon, 1942) AMR is evident when disease-causing bacteria are not eradicated following treatment with an antibiotic at a dose that would be anticipated to be effective. The outcome of AMR is continued infection, deleterious patient outcome and increased healthcare costs. (O'Neill, 2016) AMR is an increasing global problem as societies becomes more dependent on antibiotic use.

The underlying mechanisms of AMR are complex and often poorly understood. A known driver of AMR is the non-judicious use of antibiotics, which includes over prescription of inappropriate antibiotics and frequently at ineffective doses. (Morrill et al., 2016) This creates a selective environment for bacteria with AMR properties. Attempts to treat AMR-associated infections can lead to administration of increasingly higher antibiotic doses, which may elevate minimum inhibitory concentrations (MICs). (Opatowski et al., 2010) In such cases, treatment requires an alternative antibiotic to which the bacteria are susceptible, if such an antibiotic is available.

A contributing problem to AMR is the frequent involvement of microbial biofilms in human infection. (Bowler et al., 2020) Biofilms form when bacteria adhere to a surface and become embedded within self-produced extracellular polymeric substances (EPS). (Høiby, 2017) One of the most striking properties of biofilm encased bacterial cells is that they are often several 1000-fold less susceptible to antibiotics. (Mulcahy et al., 2008) The reasons for this are numerous and include the presence of EPS, which can sequester and inactivate antimicrobials, the variable activity of bacteria in different regions of the biofilm, and the presence of persister cells, which have phenotypic traits rendering them more tolerant to antibiotics. (Hall and Mah, 2017) Furthermore, biofilm bacteria can generate molecules (or indeed the involved genes themselves) that protect against antibiotics (*e.g.*, β-lactamase enzymes), and these can accumulate in the EPS matrix and protect neighbouring bacteria. (Brook, 2009)

Given the rate at which AMR develops and the high prevalence of microbial biofilms in human infection, new drugs that are not prone to AMR and are also active against biofilms must become central to the infectious disease management repertoire.

**Figure 1:**
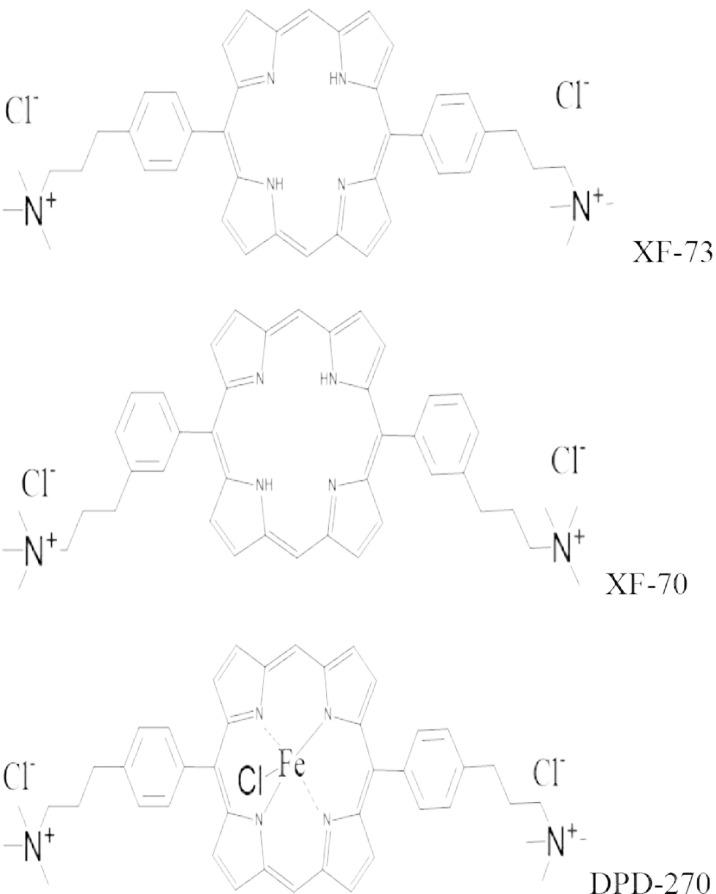
Chemical structures of the studied XF-drugs.

Recently, a new class of antibacterials, termed XF drugs, has been developed by Destiny Pharma plc, Brighton, UK (**Figure 1**). Two of these drugs (XF-73 and XF-70) have dual mechanisms of action. It has been reported that XF-73 selectively binds to bacterial cell membranes, which leads to membrane disruption and rapid loss of potassium and ATP from the cells. Consequently, inhibition of DNA, RNA and protein synthesis occurs with a loss of cell viability. (Ooi et al., 2009a) In addition to XF-73, disruption in membrane integrity is also a mechanism of action associated with both XF-70 and DPD-207 (Ooi et al., 2009b) with XF-73 and XF-70 having more pronounced initial effects on the membrane potential than DPD-207. A second, light-activated mechanism of action, is due to the presence of a porphyrin ring structure within XF-73 and XF-70 (**Figure 1**), causing release of reactive oxygen species (ROS), particularly singlet oxygen. (Maisch et al., 2005) In this process, the presence of the porphyrin structure enables the drug to act as a photosensitiser. Light absorption in the presence of oxygen results in a triplet state of the excited photosensitizer. A type I or type II reaction may then occur. The type I reaction sees production of hydrogen peroxide, superoxide radical anions, or hydroxyl radicals following electron transfer from the photosensitizer to suitable substrates. In a type II reaction, the excited photosensitizer reacts directly with molecular oxygen to produce singlet oxygen (^1^O_2_). Singlet oxygen is highly reactive resulting in oxidation of bacterial components such as molecules associated with the cell wall, membranes, or nucleic acids.

The addition of a metal ion to the centre of a porphyrin ring inactivates the production of ROS, and this is the reason why DPD-207 lacks the second light-activated antibacterial action (unpublished data).

Bacterial resistance to XF-73 has previously been studied using four strains of methicillin-resistant *Staphylococcus aureus* (MRSA). No resistance was observed over 55 passages for intrinsic XF-73 activity, which was not the case for control antibiotics (mupirocin, fusidic acid, daptomycin, retapamulin and vancomycin). (Farrell et al., 2011) Additionally, to the best of our knowledge, no bacterial resistance to ROS has been reported, suggesting that the second light-activated mechanism would also not be affected. This AMR profile highlights the significant potential for longevity of antimicrobial efficacy for XF drugs.

Antibacterial activity of XF-73 has been reported against aerobic and anaerobic Gram-positive bacteria including species of *Staphylococcus, Enterococcus* and *Streptococcus*, and isolates known to be antibiotic resistant. (Farrell et al., 2010) Antibiofilm data has been published for XF-70 and XF-73 with minimum biofilm eradication concentrations (MBECs) at 2-fold the planktonic MIC for a biofilm forming *S. aureus* isolate. (Ooi et al., 2010)

This present study further investigated the antibacterial and antibiofilm activity of XF-73, XF-70 and DPD-207 drugs. In addition, the impact of PD activity on promoting light-activated antibacterial potency for both XF-73 and XF-70 was assessed. The synergistic effects of XF drugs with conventional antibiotics were also explored, thereby highlighting the potential of XF drugs in combinational therapy.

## Materials and Methods

### Microorganisms

A total of 114 bacterial isolates were included in this study (Supplementary Data - Bacterial Isolates). Bacteria were maintained on blood agar (Fisher Scientific, UK) prior to overnight culture at 37°C in Muller Hinton Broth (MHB; Fisher Scientific).

### Antimicrobial Drugs

#### XF Drugs

XF drugs were provided by Destiny Pharma plc and re-suspended in distilled water to generate stock concentrations of 10 mg/ml. These agents were stored at 4**°**C for up to 1 week prior to use.

#### Antibiotics

Ertapenem, polymyxin B, mupirocin, retapamulin and chloramphenicol (Sigma-Aldrich, UK) were prepared as 10 mg/ml stock solutions as directed.

### Susceptibility of Bacteria to XF Drugs Using Broth Microdilution

The Minimum Inhibitory Concentration (MIC) of XF drugs was determined against test bacteria using a broth microdilution method. (Clinical and Laboratory Standards Institute, 2012) Briefly, 100 μl of XF drug (XF-73, XF-70 and DPD-207) at a range of concentrations (0 - 1024 μg/ml) in MHB was added to the wells of a 96-well microtitre plate. Overnight bacterial cultures in MHB were adjusted to a 0.5 McFarland standard and diluted 10-fold. Five μl of these cultures were added to each drug concentration to generate an inoculum *ca.* 5x10^5^ colony forming units (CFU)/ml, per well. In the case of XF-73 and XF-70, selected microtitre plates were also used to assess PD action through illumination (at a wavelength of 380-480 nm using a modified light source (Waldmann Medizintechnik, Villingen-Schwenningen, Germany) delivering 14 J/cm^2^ of light) for 15 min. All plates were subsequently incubated aerobically at 37**°**C for 16-20 h and visually analysed for bacterial growth. All tests were done in triplicate (independent biological replicates).

### Synergistic Effects of XF Drugs With Conventional Antibiotics

Synergistic effects of XF drugs and conventional antibiotics were assessed using a checkerboard assay. (Orhan et al., 2005) Antibiotic and bacterial isolate combinations were chosen based on typical use of antibiotics. Ertapenem, (Congeni, 2010) polymyxin B, mupirocin and retapamulin (Williamson et al., 2017) are antibiotics used to treat skin infections caused by *P. aeruginosa*, *S. aureus* (Burnham et al., 2016) and *E. coli.* (Johnson and Russo, 2002) As DPD-207 is not photo-dynamically activated, it lends itself well as an ophthalmic therapeutic. Therefore, DPD-207 was investigated for synergistic effects with currently used ophthalmic antibiotics, namely chloramphenicol and polymyxin B. (Robert and Adenis, 2001) As activity of both XF-73 and XF-70 might be further enhanced by PD activation, antibiotics used to treat skin and lung infections were chosen, as PD action can be used in a number of clinical situations.

Briefly, 50 μl of XF drug (XF-73, XF-70 and DPD-207) and antibiotic in MHB were added to a 96-well microtitre plate at twice the assay concentration to account for further dilutions. Overnight bacterial cultures were adjusted to a 0.5 McFarland standard and diluted 10-fold. Five μl of diluted cultures were added to each drug concentration to generate 5x10^5^ CFU/ml per well. For samples requiring PD activation (XF-73 and XF-70), plates were illuminated as previously described. All plates were then incubated at 37**°**C for 16-20 h, and visually analysed for bacterial growth. All tests were all done in triplicate (independent biological replicates). Synergy was determined using the calculation for fractional inhibitory concentration (ΣFIC) as indicated below:


$$FICindex=\frac{MIC{\left(drugA\right)}_{combinationAB}}{MIC{\left(drugA\right)}_{alone}}+\frac{MIC{\left(drugB\right)}_{combinationAB}}{MIC{\left(drugB\right)}_{alone}}$$

Based on the above formula, an FIC index of ≤ 0.5 = synergy, >0.5 - < 4 = no interaction and ≥ 4 = antagonism. (Odds, 2003)

### Susceptibility Testing of XF Drugs Against Biofilms

The minimum biofilm eradication concentration (MBEC) was determined based on methodology previously reported (Hooper et al., 2011), (Serra et al., 2018) with some minor modification. Briefly, microtitre plates containing 5x10^6^ CFU/ml bacteria in a 100-μl volume of MHB were incubated at 37**°**C for 24 h to facilitate biofilm formation. Culture medium was then removed and attached biofilms washed with PBS. XF drugs (XF-73, XF-70 and DPD-207) were added in 100 μl volumes of MHB (highest tested concentration was 1024 μg/ml). Microtitre plates were incubated at 37**°**C for 24 h. For PD activation (XF-73 and XF-70) tests, biofilms were formed as above and after addition of the drugs, the plates were illuminated as previously described for broth microdilution testing. Antibacterials were removed, and the biofilms washed with PBS, before fresh MHB was added. Mechanical disruption of the biofilms was performed by repeated pipetting followed by further incubation for 24 h at 37**°**C. The MBEC was determined based on visual comparisons with drug free controls. All tests were done in triplicate (independent biological replicates).

### Statistical Analysis

Results for MIC and MBEC for each isolate are expressed as the modal averages over the replicates for each isolate. Data for MIC and MBEC are classed with respect to values given by 2*^x^*, where *x* can be both positive (an integer) and negative (a fraction). This process might skew data and so a measure of variation that should adjust for changes in scale is used, namely, the coefficient of variation expressed as a percentage (= 100% × standard deviation/mean). Data for MIC and MBEC is linearised *via* log-transformation and the (unpaired) two-sample *t*-test applied to this log-transformed data in order to detect differences in these quantities due to PDT application. Statistical analyses presented are for specific isolates and so some caution should be used when interpreting results of statistical tests due to small sample sizes (3 replicates for each isolate).

## Results

### Susceptibility of Bacteria to XF Drugs Using Broth Microdilution

To analyse the spectrum of antibacterial activity, the MIC against 114 different bacterial isolates was determined for all three XF drugs (Supplementary Data - MIC), including with PD activation where appropriate. The results demonstrated that all 55 tested Gram-positive bacteria (*Staphylococcus aureus* n=19, *Staphylococcus cohnii* n=1, *Staphylococcus saprophyticus* n=1, *Staphylococcus epidermidis* n=1, *Staphylococcus hominis* n=1, *Staphylococcus warneri* n=4, coagulase negative *Staphylococcus* n=3, *Streptococcus gordonii* n=1, *Streptococcus mutans* n=1, *Streptococcus oralis* n=1, *Streptococcus pyogenes* n=1, *Enterococcus faecalis* n=4; *Enterococcus faecium* n=17) were susceptible to XF drugs (Supplementary Data - MIC), with MIC values for XF-73 and XF-70 lower than for DPD-207. In the case of the 59 tested Gram negative bacteria (*Acinetobacter baumannii* n=5, *Escherichia coli* n=5; *Klebsiella oxytoca* n=1; *Klebsiella pneumoniae* n=10; *Morganella morganii* n=2; *Pseudomonas aeruginosa* n=11; *Proteus mirabilis* n=7; *Providencia rettgeri* n=1; *Providencia stuartii* n=6; *Proteus vulgaris* n=1; *Serratia marcescens* n=6; *Enterobacter cloacae* n=3; *Enterobacter* sp n=1), 14 isolates were susceptible, and 14 were not susceptible to all XF-drugs without PD activation. PD enhancement of the antibacterial activity was evident for certain Gram-positive (XF-73, 47 isolates; XF-70, 55 isolates) and Gram-negative (XF-73, n=36 isolates; XF-70, n=47 isolates) bacteria. No antimicrobial effect was evident when PD was used in the absence of test agent.

**Table 1** presents the MICs for XF-73 ( ± PD), XF-70 ( ± PD) and DPD-207 against *Acinetobacter baumannii, Pseudomonas aeruginosa, Escherichia coli, S. aureus*, MRSA and *Staphylococcus hominis* isolates, which were used in further studies. PD activation significantly enhanced the potency, i.e., reduced the MIC of both XF-73 and XF-70 against the range of isolates as indicated in **Table 1**, with XF-70 typically having lower MIC values with PD activation compared to XF-73. Of the tested drugs, XF-70 was generally more effective against the Gram-negative bacteria tested.

**Table 1:** Minimum inhibitory concentrations (µg/ml) of XF drugs to selected bacteria.

Bacterial Strain	XF-73	XF-73 + PD	P-value	XF-70	XF-70 + PD	P-value	DPD-207
*A. baumannii* 5	512 (28.6%)	1 (34.6%)	<0.001	128 (0%)	0.125 (0%)	<0.001^†^	128
*P. aeruginosa* 48	512 (0%)	64 (0%)	<0.001^†^	128 (0%)	4 (0%)	<0.001^†^	No MIC observed
*E. coli* 52	512 (0%)	256 (0%)	<0.001^†^	32 (43.3%)	8 (43.3%)	0.013	512
*S. aureus* 73	1 (34.6%)	0.03 (0%)	0.005	1 (43.3%)	0.06 (0%)	0.006	4
*S. aureus* 77	2 (28.6%)	0.5 (43.3%)	0.018	1 (0%)	0.125 (0%)	<0.001^†^	2
MRSA 79	2 (43.3%)	0.125 (43.3%)	0.001	1 (0%)	0.06 (0%)	<0.001^†^	2
*S. hominis* 98	0.25 (0%)	0.015 (27.3%)	<0.001	1 (0%)	0.03 (34.6%)	0.004	2

+PD, with photodynamic therapy; MRSA, methicillin resistant S. aureus; all tests were undertaken with triplicate cultures. Figures in brackets indicate the coefficient of variation between replicates, which is expressed here as a percentage. P-values are for the (unpaired) two-sample t-test applied to log-transformed data to test for differences in MIC between groups with and without PDT; ^†^ indicates those cases where variation is zero in both groups. (Caution should be exercised when interpreting results of statistical tests due to small sample sizes; 3 replicates per group).

### Synergistic Effects of XF Drugs With Conventional Antibiotics

Potential synergistic effects of XF drugs with conventional antibiotics were assessed. XF drugs and antibiotics were combined at different concentrations and bacterial growth analysed. **Tables 2A–C** present the results of synergy assays showing that XF-73 and XF-70 had synergy with ertapenem against MRSA (isolate number 79) irrespective of whether the innate antimicrobial activity was PD enhanced. Additionally, XF-73 displayed synergy with polymyxin B against *P. aeruginosa* (isolate number 48), irrespective of whether the innate antimicrobial activity was enhanced by PD action.

**Table 2a:** Synergistic effects of XF-73 with antibiotics.

Bacterial Strain	PD	Antibiotic	Antibiotic MIC µg/ml			XF-73 MIC µg/ml				
			Alone	Combined		Alone	Combined	ΣFIC	Effect
***P. aeruginosa* 48**	–	Ertapenem	4	4		512	512	2.00	None
***P. aeruginosa* 48**	+	Ertapenem	4	2		64	32	1.00	None
***E. coli* 52**	–	Ertapenem	0.0078	0.0078		512	512	2.00	None
***E. coli* 52**	+	Ertapenem	0.0078	0.0078		256	256	2.00	None
**MRSA 79**	–	Ertapenem	256	32		2	0.03	**0.31**	**Synergy**
**MRSA 79**	+	Ertapenem	256	64		0.125	0.0078	**0.31**	**Synergy**
***P. aeruginosa* 48**	–	Polymyxin B	2	0.5		512	128	**0.50**	**Synergy**
***P. aeruginosa* 48**	+	Polymyxin B	0.5	0.125		128	8	**0.31**	**Synergy**
***E. coli* 52**	–	Polymyxin B	0.5	0.5		512	64	1.12	None
***E. coli* 52**	+	Polymyxin B	0.5	0.5		256	16	1.06	None
**MRSA 79**	–	Mupirocin	0.125	0.06		1	0.25	0.73	None
MRSA 79	+	Mupirocin	0.125	0.06		0.125	0.06	0.96	None
MRSA 79	–	Retapamulin	0.06	0.03		0.5	0.25	1.00	None
MRSA 79	+	Retapamulin	0.03	0.015		0.125	0.03	0.74	None

All tests were undertaken with triplicate cultures. PD, photodynamic therapy.

ΣFIC ≤ 0.5 = Synergy. ΣFIC > 0.5 - < 4 = No interaction. ΣFIC ≥ 4 = Antagonism.

**Table 2b:** Synergistic effects of XF-70 with antibiotics.

Bacterial Strain	PD	Antibiotic	Antibiotic MIC µg/ml		XF-70 MIC µg/ml		ΣFIC	Effect
			Alone	Combined	Alone	Combined		
***P. aeruginosa* 48**	–	Ertapenem	4	4	128	64	1.50	None
***P. aeruginosa* 48**	+	Ertapenem	4	4	4	4	2.00	None
***E. coli* 52**	–	Ertapenem	0.0078	0.0078	32	32	2.00	None
***E. coli* 52**	+	Ertapenem	0.0078	0.015	16	16	2.92	None
**MRSA 79**	–	Ertapenem	256	16	1	0.125	**0.19**	**Synergy**
**MRSA 79**	+	Ertapenem	256	64	0.06	0.0039	**0.31**	**Synergy**
***P. aeruginosa* 48**	–	Polymyxin B	0.5	0.125	128	128	1.25	None
***P. aeruginosa* 48**	+	Polymyxin B	0.5	0.25	4	0.25	0.56	None
***E. coli* 52**	–	Polymyxin B	0.5	0.25	32	16	0.51	None
***E. coli* 52**	+	Polymyxin B	0.5	0.25	8	2	0.75	None
**MRSA 79**	–	Mupirocin	0.125	0.06	1	0.125	0.61	None
**MRSA 79**	+	Mupirocin	0.125	0.03	0.015	0.0039	0.51	None
**MRSA 79**	–	Retapamulin	0.06	0.06	1	1	2.00	None
**MRSA 79**	+	Retapamulin	0.03	0.015	0.125	0.015	0.62	None

All tests were undertaken with triplicate cultures. PD, photodynamic therapy.

ΣFIC ≤ 0.5 = Synergy. ΣFIC > 0.5 - < 4 = No interaction. ΣFIC ≥ 4 = Antagonism.

**Table 2c:** Synergistic effects of DPD-207 with antibiotics.

Bacterial Strain	Antibiotic	Antibiotic MIC µg/ml		DPD-207 MIC µg/ml		ΣFIC	Effect
		Alone	Combined	Alone	Combined		
***P. aeruginosa* 48**	Chloramphenicol	64	16	512	256	0.75	No interaction
**MRSA 79**	Chloramphenicol	4	4	2	0.5	1.25	No interaction
***P. aeruginosa* 48**	Polymyxin B	0.5	0.5	>1024	64	0.75	No interaction

All tests were undertaken with triplicate cultures.

ΣFIC ≤ 0.5 = Synergy. ΣFIC > 0.5 - < 4 = No interaction. ΣFIC ≥ 4 = Antagonism.

### Susceptibility Testing of XF Drugs Against Biofilms

The effect of XF drugs against biofilms was investigated using MBEC assays. Biofilms were treated with XF drugs, and apart from DPD-207 the effects of PD action were also assessed. Following drug removal, any surviving bacteria within the biofilms were allowed to regrow. Regrowth was measured visually 24 h post treatment. The assay recorded the concentration (MBEC) at which XF drugs completely eradicated the biofilms and prevented regrowth. **Table 3** presents the MBECs of XF-drugs innately and *via* PD (apart from DPD-207). XF-73 and XF-70 had MBECs between 2-16 μg/ml, irrespective of PD activation for all Gram-positive bacteria with XF-73. No antibiofilm effect was observed against the Gram-negative isolates apart for XF-73 with PD against *Acinetobacter baumannii*, whose MBEC was enhanced from 1024 μg/ml to 128 μg/ml. As with the MIC data, MBEC values for DPD-207 were higher than for XF-70 and XF-73.

**Table 3:** Minimum biofilm eradication concentrations (MBECs) for XF-73.

XF drug	PD	MBEC (μg/ml) against test bacteria				
		*A. baumannii* 5	*S. aureus* 73	*S. aureus* 77	MRSA 79	*S. hominis* 98
**XF-73**	–	>1024 (*) (0%)	8 (0%)	8 (43.3%)	2 (43.3%)	4 (34.6%)
**XF-73**	+	>128 (0%)	8 (0%)	16 (0%)	4 (0%)	8 (43.3%)
**P-value**		<0.001^†^	N/A	0.184	0.184	0.024
**XF-70**	–	>128 (0%)	8 (0%)	8 (43.3%)	4 (0%)	4 (0%)
**XF-70**	+	>128 (0%)	8 (43.3%)	16 (0%)	8 (43.3%)	8 (43.3%)
**P-value**		N/A	0.423	0.184	0.057	0.057
**DPD-207**	–	>128 (0%)	128 (0%)	128 (0%)	128 (0%)	128 (0%)

All tests were undertaken in triplicate. * cannot measure COV due to no minimum value. Figures in brackets indicate the coefficient of variation between replicates, which is expressed here as a percentage. P-values are for the (unpaired) two-sample t-test applied to log-transformed data to test for differences in MBEC between groups with and without PDT. (^†^ indicates those cases where variation is zero in both groups, t-values tend to infinity, and so P-values are assumed explicitly to tend to zero here. However, caution should be exercised when interpreting results of all statistical tests due to small sample sizes; 3 per group. N/A shows those cases where the t-value cannot be estimated).

## Discussion

As the global burden of AMR increases, (O'Neill, 2016) development of new antimicrobials that are effective against clinically relevant bacteria is imperative. Destiny Pharma plc have developed a platform of XF drugs, with an intrinsic mechanism of action which involves the binding and disruption of bacterial membrane integrity, resulting in the rapid loss of potassium and ATP from the cells, the inhibition of DNA, RNA and protein synthesis and loss of viability, without lysis of the bacterial cell (Ooi et al., 2009a) In this present study, which expands our knowledge of XF drugs, susceptibility was highest for Gram-positive bacteria, with Gram-negative bacteria being less susceptible. This finding agrees with previous research (Farrell et al., 2010) and was expected, given that the outer lipopolysaccharide layer in the Gram-negative cell wall can shield bacterial cells from exogenous agents. (Livermore, 1990) Importantly, antimicrobial activity was found against Gram-positive isolates currently resistant to conventional antibiotics (isolates 53-72 and 79; Supplementary Data – Bacterial Isolates). XF-73 and XF-70 were potent against all Gram-positive isolates tested and this activity could be significantly enhanced (lowering MICs) with PD activation. Generally, the XF drugs exhibited higher MICs against Gram-negative isolates, however PD significantly enhanced potency, particularly for XF-70. Generally, PD did not enhance activity against bacterial isolates within biofilm, with the exception of XF-73 against *A.baumanii* biofilm. MICs for DPD-207 were higher than the corresponding MICs for XF-70 and XF-73.

Enhancement of antibacterial effects using PD action was investigated with XF-73 and XF-70, as both contain a porphyrin ring which facilitate the release of ROS after PD activation, a second antimicrobial mechanism of action. (Maisch et al., 2005)

As DPD-207 was specifically designed to have no PD action it was not investigated in these particular studies. Our findings demonstrated that PD activation could lower the MIC of XF-73 and XF-70 against certain isolates, and thus increased their potency and bacterial susceptibility. This might be a useful approach where topical prophylaxis or treatment of skin infections, such as with burns or chronic wounds, is required, and PD action can easily be applied to the affected area, also allowing lower doses of XF drugs to be used.

Synergistic effects of XF drugs with conventional antibiotics were also evident, which may also allow combination therapy with lower doses of each drug. XF drugs cause bacterial membrane disruption, (Ooi et al., 2009b) which could enhance penetration of other antibiotics into bacterial cells. Synergistic effects of XF-73 and XF-70 were therefore investigated with these antibiotics, with and without PD activation. Our finding that XF-73 and XF-70 had synergy with ertapenem against MRSA gives promise for a combination therapy to treat drug-resistant skin infections using lower doses of ertapenem, reducing the risk of potential side-effects or further AMR development. Synergy between polymyxin B and XF-73 was also evident and independent of PD treatment, suggesting that the effect was due to the intrinsic antimicrobial mechanism of action. This finding demonstrated that light delivery to a treatment site would not comprise synergistic outcome and that the dual antimicrobial activity of XF-70 and XF-73 could be used in combination with synergistic effects. The combination of DPD-207 with chloramphenicol and polymixin B was assessed against *P. aeruginosa* and *S. aureus*, which are known causative agents of ophthalmic infections. (Lorenzo, 2019) No synergy was observed with the tested antibiotics.

Antibiofilm properties of XF-drugs, including PD effect, were investigated using an MBEC assay. As XF-73 and XF-70 had previously been shown to be effective against *S. aureus* biofilms, (Ooi et al., 2010) three different *S. aureus* isolates were examined, including an MRSA strain. A further *Staphylococcus* species was chosen to investigate whether similar effects occurred across the *Staphylococcus* genus. As the MIC of the Gram-negative *A. baumannii* (isolate number 5) had dramatically been reduced using PD activation, this isolate was also chosen to investigate whether similar effects occurred with biofilms.

XF drugs had antibiofilm effects from concentrations as low as 2 μg/ml against the tested Gram-positive bacteria. This agreed with previously published data showing an MBEC of 2 μg/ml against *S. aureus* SH1000 for both XF-73 and XF-70. (Ooi et al., 2010) MBECs of 2 μg/ml were evident with *S. aureus* isolate 79 for XF-73; for both XF-73 and XF-70 in the absence of PD MBECs of 8 μg/ml were found with isolates 73 and 77. The higher MBEC (8 μg/ml) was two-fold above that found in a previous study. (Ooi et al., 2010) This previous study used a Calgary biofilm device to grow grown on pegs, whereas our study used the base of wells in a microtiter plate as the surface to grow the biofilms. It is possible that this difference in methodology together with testing a different strain explains the small variation in MBECs observed across the two studies.

While we found that PD action reduced the MIC of XF-73 and XF-70 against some of the isolates investigated *i.e.*, increased their potency, PD activation of these XF drugs had no effect on the MBEC values in this model (other than XF-73 PD against *A. baumannii)*. The values either remained the same or increased by two-fold. This increase was likely due to experimental variation and was not deemed significant. We speculate several reasons for the lack of PD effect on biofilms in this model. Firstly, the light may not have been able to penetrate the biofilms, hence no further antibiofilm effects were observed after PD activation. Secondly, lower oxygen levels within *in vitro S. aureus* biofilms, (Kiamco et al., 2018) may reduce ROS generation. As ROS production is the mechanism by which PD action acts, (Maisch et al., 2005) an anaerobic environment would limit such effects. Further work is required to determine whether alternative methods of PD delivery can enhance the antibacterial effects.

XF-73 and XF-70 were found to be active against Gram-positive biofilms with slightly higher MBECs than the corresponding MICs for the planktonic forms. This is in line with the previous study by Ooi *et al*., which demonstrated an MBEC of 2 μg/ml compared to an MIC of 1 μg/ml against *S. aureus* SH1000 for both XF73 and XF-70. (Ooi et al., 2010) This was a surprising and potentially beneficial finding, as MBEC values are often found to be much higher, up to several 1000-fold the MIC values. (Ceri et al., 1999; Olson et al., 2002; Fux et al., 2004; Nishimura et al., 2006; Girard et al., 2010; Castaneda et al., 2016). This demonstrates the potent antibiofilm effects of the XF drugs.

Overall, these studies provide evidence that the XF drug platform was effective against a wide-range of bacteria, including those resistant to conventional antibiotics, with effects enhanced by the PD mechanism of action. Additionally, XF drugs also displayed synergy with specific conventional antibiotics. Initial biofilm investigations also showed promise, with observations of antibiofilm effects of XF drugs with the MBEC assay. Future studies will evaluate the effects of XF drugs on more complex biofilms, generated using shear force and flow systems.

## Data Availability Statement

The original contributions presented in the study are included in the article/**Supplementary Material**. Further inquiries can be directed to the corresponding author.

## Author Contributions

DW and EB-D conceived the experiments. EB-D performed the experiments. EB-D, DW, DF, WL, DH, and WR-W reviewed the data. EB-D and DW wrote the manuscript. All authors contributed to the article and approved the submitted version.

## Funding

Funding was provided by the Department of Health and Social Care through Innovate UK grant number: 104987.

## Conflict of Interest

Authors WR-W, DH, and WL, are employed by Destiny Pharma plc.

The remaining authors declare that the research was conducted in the absence of any commercial or financial relationships that could be construed as a potential conflict of interest.

## Publisher’s Note

All claims expressed in this article are solely those of the authors and do not necessarily represent those of their affiliated organizations, or those of the publisher, the editors and the reviewers. Any product that may be evaluated in this article, or claim that may be made by its manufacturer, is not guaranteed or endorsed by the publisher.
