# Supplementary material for: Antibacterial and Antibiofilm Potency of XF Drugs, Impact of Photodynamic Activation and Synergy With Antibiotics
Source: Front Cell Infect Microbiol. 2022 Jun 30;12:904465. doi: 10.3389/fcimb.2022.904465 (PMC9279914; doi:10.3389/fcimb.2022.904465)
Supplement: Supplementary file 1 [file Table_1.docx]

**Supplementary Material**

Table A1: Bacterial isolates used for susceptibility testing

| \| Isolate number \| Bacterial species \| Gram reaction \| Source \| \| --- \| --- \| --- \| --- \| \| 5 \| *Acinetobacter baumannii* \| negative \| Urine samples from hospital wards in Wales \| \| 6 \| *Acinetobacter baumannii* \| negative \| Urine samples from hospital wards in Wales \| \| 7 \| *Acinetobacter baumannii* \| negative \| Urine samples from hospital wards in Wales \| \| 8 \| *Acinetobacter baumannii* \| negative \| Urine samples from hospital wards in Wales \| \| 113 \| *Acinetobacter baumannii* \| negative \| Faecal samples \| \| 11 \| *Enterobacter cloacae* \| negative \| Urine samples from hospital wards in Wales \| \| 12 \| *Enterobacter cloacae* \| negative \| Urine samples from hospital wards in Wales \| \| 13 \| *Enterobacter cloacae* \| negative \| Urine samples from hospital wards in Wales \| \| 9 \| *Escherichia coli* \| negative \| Urine samples from hospital wards in Wales \| \| 16 \| *Escherichia coli* \| negative \| Urine samples from hospital wards in Wales \| \| 17 \| *Escherichia coli* \| negative \| Urine samples from hospital wards in Wales \| \| 18 \| *Escherichia coli* \| negative \| Urine samples from hospital wards in Wales \| \| 52 \| *Escherichia coli* \| negative \| NCTC 12923 \| \| 41 \| *Klebsiella oxytoca* \| negative \| Urinary tract infections \| \| 29 \| *Klebsiella pneumonia* \| negative \| Urinary tract infections \| \| 1 \| *Klebsiella pneumoniae* \| negative \| Urine samples from hospital wards in Wales \| \| 2 \| *Klebsiella pneumoniae* \| negative \| Urine samples from hospital wards in Wales \| \| 3 \| *Klebsiella pneumoniae* \| negative \| Urine samples from hospital wards in Wales \| \| 4 \| *Klebsiella pneumoniae* \| negative \| Urine samples from hospital wards in Wales \| \| 10 \| *Klebsiella pneumoniae* \| negative \| Urine samples from hospital wards in Wales \| \| 23 \| *Klebsiella pneumoniae* \| negative \| Urinary tract infections \| \| 32 \| *Klebsiella pneumoniae* \| negative \| Urinary tract infections \| \| 112 \| *Klebsiella pneumoniae* \| negative \| Faecal samples \| \| 114 \| *Klebsiella pneumoniae* \| negative \| Faecal samples \| \| 39 \| *Morganella morganii* \| negative \| Urinary tract infections \| \| 45 \| *Morganella morganii* \| negative \| Urinary tract infections \| \| 24 \| *Proteus mirabilis* \| negative \| Urinary tract infections \| \| 30 \| *Proteus mirabilis* \| negative \| Urinary tract infections \| \| 31 \| *Proteus mirabilis* \| negative \| Urinary tract infections \| \| 35 \| *Proteus mirabilis* \| negative \| Urinary tract infections \| \| 37 \| *Proteus mirabilis* \| negative \| Urinary tract infections \| \| 43 \| *Proteus mirabilis* \| negative \| Urinary tract infections \| \| 44 \| *Proteus mirabilis* \| negative \| Urinary tract infections \| \| 25 \| *Proteus vulgaris* \| negative \| Urinary tract infections \| \| 42 \| *Providencia rettgeri* \| negative \| Urinary tract infections \| \| 22 \| *Providencia stuartii* \| negative \| Urinary tract infections \| \| 28 \| *Providencia stuartii* \| negative \| Urinary tract infections \| \| 34 \| *Providencia stuartii* \| negative \| Urinary tract infections \| \| 36 \| *Providencia stuartii* \| negative \| Urinary tract infections \| \| 38 \| *Providencia stuartii* \| negative \| Urinary tract infections \| \| 40 \| *Providencia stuartii* \| negative \| Urinary tract infections \| \| 14 \| *Pseudomonas aeruginosa* \| negative \| Urine samples from hospital wards in Wales \| \| 15 \| *Pseudomonas aeruginosa* \| negative \| Urine samples from hospital wards in Wales \| \| 19 \| *Pseudomonas aeruginosa* \| negative \| Urine samples from hospital wards in Wales \| \| 20 \| *Pseudomonas aeruginosa* \| negative \| Urine samples from hospital wards in Wales \| \| 46 \| *Pseudomonas aeruginosa* \| negative \| Chronic wound \| \| 47 \| *Pseudomonas aeruginosa* \| negative \| Chronic wound \| \| 48 \| *Pseudomonas aeruginosa* \| negative \| Chronic wound \| \| 49 \| *Pseudomonas aeruginosa* \| negative \| Sputum of a cystic fibrosis patient \| \| 50 \| *Pseudomonas aeruginosa* \| negative \| ATCC 15692 \| \| 51 \| *Pseudomonas aeruginosa* \| negative \| NCTC 10662 \| \| 111 \| *Pseudomonas aeruginosa* \| negative \| Faecal samples \| \| 21 \| *Serratia marcescens* \| negative \| Urinary tract infections \| \| 26 \| *Serratia marcescens* \| negative \| Urinary tract infections \| \| 27 \| *Serratia marcescens* \| negative \| Urinary tract infections \| \| 33 \| *Serratia marcescens* \| negative \| Urinary tract infections \| \| *75* \| *Serratia marcescens* \| negative \| Urinary tract infections \| \| 76 \| *Serratia marcescens* \| negative \| Urinary tract infections \| \| 81 \| coagulase-negative (CN) *Staphylococcus* \| positive \| Chronic wound \| \| 86 \| *Enterococcus faecalis* \| positive \| Oral Squamous Cell Carcinoma Mucosal Tissue \| \| 87 \| *Enterococcus faecalis* \| positive \| NCTC 775 \| \| 59 \| *Enterococcus faecalis* (VRE) \| positive \| Urine sample from a patient on Nephrology ward \| \| 69 \| *Enterococcus faecalis* (VRE) \| positive \| Blood sample from an ICU patient at UHW \| \| 70 \| *Enterococcus faecalis* (VRE) \| positive \| Sputum sample from a patient on nephrology ward \| \| 53 \| *Enterococcus faecium* (VRE) \| positive \| Blood sample from a patient on haematology ward \| \| 54 \| *Enterococcus faecium* (VRE) \| positive \| Blood sample from an ICU patient at UHW \| \| 55 \| *Enterococcus faecium* (VRE) \| positive \| Blood sample from an ICU patient at UHW \| \| 56 \| *Enterococcus faecium* (VRE) \| positive \| Blood sample from a patient on haematology ward \| \| 57 \| *Enterococcus faecium* (VRE) \| positive \| Blood sample from an ICU patient at UHW \| \| 58 \| *Enterococcus faecium* (VRE) \| positive \| Blood sample from a patient on Nephrology ward \| \| 60 \| *Enterococcus faecium* (VRE) \| positive \| Blood sample from an ICU patient at UHW \| \| 61 \| *Enterococcus faecium* (VRE) \| positive \| Blood sample from an ICU patient at UHW \| \| 62 \| *Enterococcus faecium* (VRE) \| positive \| Blood sample from an ICU patient at UHW \| \| 63 \| *Enterococcus faecium* (VRE) \| positive \| Blood sample from an ICU patient at UHW \| \| 64 \| *Enterococcus faecium* (VRE) \| positive \| Blood sample from an ICU patient at UHW \| \| 65 \| *Enterococcus faecium* (VRE) \| positive \| Blood sample from an ICU patient at UHW \| \| 66 \| *Enterococcus faecium* (VRE) \| positive \| Bile specimen from a patient on Nephrology ward \| \| 67 \| *Enterococcus faecium* (VRE) \| positive \| Blood sample from a patient on Haematology ward \| \| 68 \| *Enterococcus faecium* (VRE) \| positive \| Dialysis fluid sample from a patient on Nephrology ward \| \| 71 \| *Enterococcus faecium* (VRE) \| positive \| Dialysis fluid sample from a patient on Nephrology ward \| \| 72 \| *Enterococcus faecium* (VRE) \| positive \| Blood sample from an ICU patient at UHW \| \| 82 \| MR-CNS \| positive \| Chronic wound \| \| 79 \| Methicillin resistant *Staphylococcus aureus* \| positive \| Chronic wound \| \| 80 \| *Staphylococcus aureus* \| positive \| Chronic wound \| \| 73 \| *Staphylococcus aureus* \| positive \| Urinary tract infections \| \| 74 \| *Staphylococcus aureus* \| positive \| Urinary tract infections \| \| 77 \| *Staphylococcus aureus* \| positive \| Urinary tract infections \| \| 78 \| *Staphylococcus aureus* \| positive \| Urinary tract infections \| \| 89 \| *Staphylococcus aureus* \| positive \| NCTC 6571 \| \| 90 \| *Staphylococcus aureus* \| positive \| Chronic wound \| \| 93 \| *Staphylococcus aureus* \| positive \| Oral Squamous Cell Carcinoma Mucosal Tissue \| \| 97 \| *Staphylococcus aureus* \| positive \| Oral Squamous Cell Carcinoma Mucosal Tissue \| \| 100 \| *Staphylococcus aureus* \| positive \| Faecal samples \| \| 101 \| *Staphylococcus aureus* \| positive \| Faecal samples \| \| 102 \| *Staphylococcus aureus* \| positive \| Faecal samples \| \| 103 \| *Staphylococcus aureus* \| positive \| Faecal samples \| \| 104 \| *Staphylococcus aureus* \| positive \| Faecal samples \| \| 105 \| *Staphylococcus aureus* \| positive \| Faecal samples \| \| 106 \| *Staphylococcus aureus* \| positive \| Faecal samples \| \| 107 \| *Staphylococcus aureus* \| positive \| Faecal samples \| \| 108 \| *Staphylococcus aureus* \| positive \| Faecal samples \| \| 109 \| *Staphylococcus aureus* \| positive \| Faecal samples \| \| 110 \| *Staphylococcus aureus* \| positive \| Faecal samples \| \| 92 \| *Staphylococcus cohnii* \| positive \| Oral Squamous Cell Carcinoma Mucosal Tissue \| \| 98 \| *Staphylococcus hominis* \| positive \| Oral Squamous Cell Carcinoma Mucosal Tissue \| \| 91 \| *Staphylococcus saprophyticus* \| positive \| Oral Squamous Cell Carcinoma Mucosal Tissue \| \| 94 \| *Staphylococcus warneri* \| positive \| Oral Squamous Cell Carcinoma Mucosal Tissue \| \| 95 \| *Staphylococcus warneri* \| positive \| Oral Squamous Cell Carcinoma Mucosal Tissue \| \| 96 \| *Staphylococcus warneri* \| positive \| Oral Squamous Cell Carcinoma Mucosal Tissue \| \| 99 \| *Staphylococcus warneri* \| positive \| Oral Squamous Cell Carcinoma Mucosal Tissue \| \| 85 \| *Streptococcus gordonii* \| positive \| Oral Squamous Cell Carcinoma Mucosal Tissue \| \| 83 \| *Streptococcus mutans* \| positive \| Oral Squamous Cell Carcinoma Mucosal Tissue \| \| 84 \| *Streptococcus oralis* \| positive \| Oral Squamous Cell Carcinoma Mucosal Tissue \| \| 88 \| *Streptococcus pyogenes* \| positive \| NCTC 8198 \| |  |
| --- | --- | --- | --- | --- | --- | --- | --- | --- | --- | --- | --- | --- | --- | --- | --- | --- | --- | --- | --- | --- | --- | --- | --- | --- | --- | --- | --- | --- | --- | --- | --- | --- | --- | --- | --- | --- | --- | --- | --- | --- | --- | --- | --- | --- | --- | --- | --- | --- | --- | --- | --- | --- | --- | --- | --- | --- | --- | --- | --- | --- | --- | --- | --- | --- | --- | --- | --- | --- | --- | --- | --- | --- | --- | --- | --- | --- | --- | --- | --- | --- | --- | --- | --- | --- | --- | --- | --- | --- | --- | --- | --- | --- | --- | --- | --- | --- | --- | --- | --- | --- | --- | --- | --- | --- | --- | --- | --- | --- | --- | --- | --- | --- | --- | --- | --- | --- | --- | --- | --- | --- | --- | --- | --- | --- | --- | --- | --- | --- | --- | --- | --- | --- | --- | --- | --- | --- | --- | --- | --- | --- | --- | --- | --- | --- | --- | --- | --- | --- | --- | --- | --- | --- | --- | --- | --- | --- | --- | --- | --- | --- | --- | --- | --- | --- | --- | --- | --- | --- | --- | --- | --- | --- | --- | --- | --- | --- | --- | --- | --- | --- | --- | --- | --- | --- | --- | --- | --- | --- | --- | --- | --- | --- | --- | --- | --- | --- | --- | --- | --- | --- | --- | --- | --- | --- | --- | --- | --- | --- | --- | --- | --- | --- | --- | --- | --- | --- | --- | --- | --- | --- | --- | --- | --- | --- | --- | --- | --- | --- | --- | --- | --- | --- | --- | --- | --- | --- | --- | --- | --- | --- | --- | --- | --- | --- | --- | --- | --- | --- | --- | --- | --- | --- | --- | --- | --- | --- | --- | --- | --- | --- | --- | --- | --- | --- | --- | --- | --- | --- | --- | --- | --- | --- | --- | --- | --- | --- | --- | --- | --- | --- | --- | --- | --- | --- | --- | --- | --- | --- | --- | --- | --- | --- | --- | --- | --- | --- | --- | --- | --- | --- | --- | --- | --- | --- | --- | --- | --- | --- | --- | --- | --- | --- | --- | --- | --- | --- | --- | --- | --- | --- | --- | --- | --- | --- | --- | --- | --- | --- | --- | --- | --- | --- | --- | --- | --- | --- | --- | --- | --- | --- | --- | --- | --- | --- | --- | --- | --- | --- | --- | --- | --- | --- | --- | --- | --- | --- | --- | --- | --- | --- | --- | --- | --- | --- | --- | --- | --- | --- | --- | --- | --- | --- | --- | --- | --- | --- | --- | --- | --- | --- | --- | --- | --- | --- | --- | --- | --- | --- | --- | --- | --- | --- | --- | --- | --- | --- | --- | --- | --- | --- | --- | --- | --- | --- | --- | --- | --- | --- | --- | --- | --- | --- | --- | --- | --- | --- | --- | --- | --- | --- | --- | --- | --- | --- | --- | --- | --- | --- | --- | --- | --- | --- | --- | --- | --- | --- | --- | --- | --- | --- | --- | --- | --- | --- | --- | --- | --- | --- | --- | --- | --- | --- | --- | --- | --- | --- | --- | --- | --- | --- | --- |
| *VRE, vancomycin resistant enterococcus*  Table A2: Minimum inhibitory concentration (MIC) values for tested isolates |  |
| \| Isolate Number \| Bacterial  Species \| Gram reaction \| XF-73 μg/ml \| XF-73 +PDT μg/ml \| XF-70 μg/ml \| XF-70 +PDT μg/ml \| DPD-207  μg/ml \| \| --- \| --- \| --- \| --- \| --- \| --- \| --- \| --- \| \| 7 \| *A. baumannii* \| negative \| 256 \| 2 \| 64 \| 0.25 \| 512 \| \| 5 \| *A. baumannii* \| negative \| 512 \| 1 \| 128 \| 0.125 \| 128 \| \| 6 \| *A. baumannii* \| negative \| >1024 \| 32 \| 64 \| 1 \| >1024 \| \| 8 \| *A. baumannii* \| negative \| 512 \| 8 \| 64 \| 0.25 \| 256 \| \| 113 \| *A. baumannii* \| negative \| 512 \| 4 \| 16 \| 0.25 \| 256 \| \| 11 \| *E. cloacae* \| negative \| 512 \| 512 \| 64 \| 32 \| 512 \| \| 12 \| *E. cloacae* \| negative \| >1024 \| 512 \| 64 \| 32 \| 512 \| \| 13 \| *E. cloacae* \| negative \| >1024 \| 512 \| 64 \| 32 \| 512 \| \| 18 \| *E. coli* \| negative \| 128 \| 64 \| 32 \| 16 \| 256 \| \| 9 \| *E. coli* \| negative \| 256 \| 128 \| 16 \| 16 \| 256 \| \| 52 \| *E. coli* \| negative \| 512 \| 256 \| 32 \| 8 \| 512 \| \| 16 \| *E. coli* \| negative \| >1024 \| 128 \| 16 \| 16 \| 128 \| \| 17 \| *E. coli* \| negative \| 512 \| 128 \| 32 \| 16 \| 256 \| \| 101 \| *Enterobacter* \| negative \| 512 \| 512 \| 64 \| 16 \| 512 \| \| 41 \| *K. oxytoca* \| negative \| 512 \| 512 \| 64 \| 32 \| >1024 \| \| 112 \| *K. pneumoniae* \| negative \| 256 \| 256 \| 64 \| 16 \| 512 \| \| 1 \| *K. pneumoniae* \| negative \| >1024 \| >1024 \| 128 \| 64 \| 512 \| \| 2 \| *K. pneumoniae* \| negative \| >1024 \| >1024 \| 64 \| 64 \| 512 \| \| 3 \| *K. pneumoniae* \| negative \| >1024 \| 512 \| 64 \| 64 \| >1024 \| \| 4 \| *K. pneumoniae* \| negative \| >1024 \| 512 \| 64 \| 64 \| >1024 \| \| 10 \| *K. pneumoniae* \| negative \| >1024 \| 256 \| 64 \| 32 \| 512 \| \| 23 \| *K. pneumoniae* \| negative \| 512 \| 512 \| 64 \| 32 \| 512 \| \| 29 \| *K. pneumoniae* \| negative \| 512 \| 512 \| 64 \| 32 \| >1024 \| \| 32 \| *K. pneumoniae* \| negative \| 512 \| 256 \| 64 \| 32 \| >1024 \| \| 114 \| *K. pneumoniae* \| negative \| 512 \| 512 \| 32 \| 16 \| >1024 \| \| 39 \| *M. morganii* \| negative \| >1024 \| >1024 \| 256 \| 64 \| >1024 \| \| 45 \| *M. morganii* \| negative \| >1024 \| 256 \| 16 \| 4 \| 512 \| \| 20 \| *P. aeruginosa* \| negative \| 256 \| 128 \| 128 \| 64 \| 512 \| \| 14 \| *P. aeruginosa* \| negative \| >1024 \| 512 \| 64 \| 64 \| 512 \| \| 15 \| *P. aeruginosa* \| negative \| >1024 \| 512 \| 64 \| 64 \| 512 \| \| 19 \| *P. aeruginosa* \| negative \| >1024 \| 512 \| 128 \| 64 \| 256 \| \| 46 \| *P. aeruginosa* \| negative \| 512 \| 256 \| 128 \| 4 \| >1024 \| \| 47 \| *P. aeruginosa* \| negative \| 512 \| 256 \| 128 \| 16 \| 512 \| \| 48 \| *P. aeruginosa* \| negative \| 512 \| 64 \| 128 \| 4 \| >1024 \| \| 49 \| *P. aeruginosa* \| negative \| >1024 \| 128 \| 512 \| 64 \| 512 \| \| 50 \| *P. aeruginosa* \| negative \| 512 \| 512 \| 128 \| 64 \| >1024 \| \| 51 \| *P. aeruginosa* \| negative \| >1024 \| 512 \| 64 \| 64 \| 512 \| \| 111 \| *P. aeruginosa* \| negative \| 512 \| 256 \| 64 \| 64 \| >1024 \| \| 24 \| *P. mirabilis* \| negative \| >1024 \| 8 \| >1024 \| 512 \| 512 \| \| 30 \| *P. mirabilis* \| negative \| >1024 \| >1024 \| >1024 \| 64 \| >1024 \| \| 31 \| *P. mirabilis* \| negative \| >1024 \| >1024 \| >1024 \| 256 \| >1024 \| \| 35 \| *P. mirabilis* \| negative \| >1024 \| 512 \| >1024 \| 256 \| >1024 \| \| 37 \| *P. mirabilis* \| negative \| >1024 \| >1024 \| >1024 \| 128 \| >1024 \| \| 43 \| *P. mirabilis* \| negative \| >1024 \| 512 \| >1024 \| 256 \| >1024 \| \| 44 \| *P. mirabilis* \| negative \| >1024 \| 512 \| >1024 \| 256 \| >1024 \| \| 42 \| *P. rettgeri* \| negative \| >1024 \| >1024 \| >1024 \| 512 \| >1024 \| \| 22 \| *P. stuartii* \| negative \| >1024 \| 512 \| >1024 \| 512 \| 512 \| \| 28 \| *P. stuartii* \| negative \| >1024 \| >1024 \| >1024 \| 512 \| >1024 \| \| 34 \| *P. stuartii* \| negative \| 512 \| 256 \| >1024 \| 256 \| >1024 \| \| 36 \| *P. stuartii* \| negative \| 512 \| 128 \| >1024 \| 256 \| >1024 \| \| 38 \| *P. stuartii* \| negative \| >1024 \| 512 \| >1024 \| 16 \| >1024 \| \| 40 \| *P. stuartii* \| negative \| >1024 \| 512 \| 512 \| 16 \| >1024 \| \| 25 \| *P. vulgaris* \| negative \| 512 \| 512 \| 32 \| 16 \| >1024 \| \| 21 \| *S. marcescens* \| negative \| >1024 \| >1024 \| >1024 \| 512 \| 512 \| \| 26 \| *S. marcescens* \| negative \| >1024 \| >1024 \| >1024 \| 512 \| >1024 \| \| 27 \| *S. marcescens* \| negative \| >1024 \| 128 \| 512 \| 512 \| >1024 \| \| 33 \| *S. marcescens* \| negative \| >1024 \| >1024 \| >1024 \| 256 \| >1024 \| \| 75 \| *S. marcescens* \| negative \| 512 \| 4 \| 512 \| 0.125 \| >1024 \| \| 76 \| *S. marcescens* \| negative \| >1024 \| 256 \| >1024 \| 4 \| >1024 \| \| 81 \| CNS \| positive \| 1 \| 0.125 \| 1 \| 0.0078 \| 4 \| \| 69 \| *E. faecalis* \| positive \| 0.25 \| 0.125 \| 1 \| 0.0019 \| 4 \| \| 87 \| *E. faecalis* \| positive \| 0.5 \| 0.125 \| 1 \| 0.125 \| 2 \| \| 59 \| *E. faecalis* \| positive \| 1 \| 0.25 \| 2 \| 0.06 \| 16 \| \| 70 \| *E. faecalis* \| positive \| 2 \| 0.25 \| 1 \| 0.0019 \| 8 \| \| 55 \| *E. faecium* \| positive \| 0.25 \| 0.125 \| 1 \| 0.125 \| 2 \| \| 57 \| *E. faecium* \| positive \| 0.25 \| 0.25 \| 0.5 \| 0.125 \| 2 \| \| 60 \| *E. faecium* \| positive \| 0.25 \| 0.25 \| 1 \| 0.125 \| 2 \| \| 64 \| *E. faecium* \| positive \| 0.25 \| 0.125 \| 1 \| 0.5 \| 2 \| \| 67 \| *E. faecium* \| positive \| 0.25 \| 0.25 \| 1 \| 0.06 \| 4 \| \| 68 \| *E. faecium* \| positive \| 0.25 \| 0.25 \| 1 \| 0.003 \| 4 \| \| 53 \| *E. faecium* \| positive \| 0.5 \| 0.25 \| 1 \| 0.125 \| 4 \| \| 56 \| *E. faecium* \| positive \| 0.5 \| 1 \| 1 \| 0.125 \| 1 \| \| 58 \| *E. faecium* \| positive \| 0.5 \| 0.25 \| 2 \| 0.25 \| 4 \| \| 61 \| *E. faecium* \| positive \| 0.5 \| 0.25 \| 1 \| 0.125 \| 2 \| \| 62 \| *E. faecium* \| positive \| 0.5 \| 0.06 \| 1 \| 0.03 \| 2 \| \| 63 \| *E. faecium* \| positive \| 0.5 \| 0.25 \| 1 \| 0.06 \| 2 \| \| 66 \| *E. faecium* \| positive \| 0.5 \| 0.25 \| 2 \| 0.06 \| 8 \| \| 71 \| *E. faecium* \| positive \| 0.5 \| 0.06 \| 1 \| 0.25 \| 4 \| \| 54 \| *E. faecium* \| positive \| 1 \| 1 \| 0.5 \| 0.06 \| 4 \| \| 65 \| *E. faecium* \| positive \| 2 \| 0.125 \| 1 \| 0.5 \| 2 \| \| 72 \| *E. faecium* \| positive \| 2 \| 0.125 \| 1 \| 0.125 \| 2 \| \| 82 \| MR-CNS \| positive \| 0.25 \| 0.125 \| 1 \| 0.03 \| 0.5 \| \| 79 \| MRSA \| positive \| 2 \| 0.125 \| 1 \| 0.06 \| 2 \| \| 80 \| MS-CNS \| positive \| 1 \| 0.125 \| 1 \| 0.015 \| 2 \| \| 97 \| *S. aureus* \| positive \| 0.25 \| 0.125 \| 2 \| 0.015 \| 2 \| \| 103 \| *S. aureus* \| positive \| 0.25 \| 0.0019 \| 1 \| 0.0019 \| 2 \| \| 105 \| *S. aureus* \| positive \| 0.25 \| 0.06 \| 1 \| 0.0019 \| 2 \| \| 106 \| *S. aureus* \| positive \| 0.25 \| 0.125 \| 1 \| 0.015 \| 8 \| \| 107 \| *S. aureus* \| positive \| 0.25 \| 0.125 \| 1 \| 0.125 \| 1 \| \| 108 \| *S. aureus* \| positive \| 0.25 \| 0.0078 \| 2 \| 0.003 \| 4 \| \| 109 \| *S. aureus* \| positive \| 0.25 \| 0.125 \| 1 \| 0.003 \| 2 \| \| 110 \| *S. aureus* \| positive \| 0.25 \| 0.06 \| 1 \| 0.003 \| 1 \| \| 74 \| *S. aureus* \| positive \| 0.5 \| 0.06 \| 0.5 \| 0.125 \| 2 \| \| 89 \| *S. aureus* \| positive \| 0.5 \| 0.06 \| 1 \| 0.003 \| 2 \| \| 90 \| *S. aureus* \| positive \| 0.5 \| 0.06 \| 1 \| 0.0078 \| 1 \| \| 73 \| *S. aureus* \| positive \| 1 \| 0.03 \| 1 \| 0.06 \| 4 \| \| 100 \| *S. aureus* \| positive \| 1 \| 0.125 \| 1 \| 0.015 \| 4 \| \| 77 \| *S. aureus* \| positive \| 2 \| 0.5 \| 1 \| 0.125 \| 2 \| \| 78 \| *S. aureus* \| positive \| 2 \| 0.25 \| 1 \| 0.015 \| 2 \| \| 93 \| *S. aureus* \| positive \| 2 \| 0.015 \| 1 \| 0.003 \| 2 \| \| 102 \| *S. aureus* \| positive \| 2 \| 0.03 \| 2 \| 0.015 \| 2 \| \| 104 \| *S. aureus* \| positive \| 2 \| 0.25 \| 2 \| 0.003 \| 8 \| \| 92 \| *S. cohnii* \| positive \| 0.06 \| 0.03 \| 0.5 \| 0.03 \| 0.5 \| \| 85 \| *S. epidermidis* \| positive \| 0.25 \| 0.125 \| 1 \| 0.06 \| 2 \| \| 86 \| *S. gordonii* \| positive \| 2 \| 0.0019 \| 2 \| 0.0019 \| 2 \| \| 98 \| *S. hominis* \| positive \| 0.25 \| 0.015 \| 1 \| 0.03 \| 2 \| \| 83 \| *S. mutans* \| positive \| 0.015 \| 1 \| 0.5 \| 0.003 \| 1 \| \| 84 \| *S. oralis* \| positive \| 0.06 \| 0.03 \| 0.5 \| 0.0019 \| 2 \| \| 88 \| *S. pyogenes* \| positive \| 0.06 \| 0.03 \| 0.5 \| 0.03 \| 0.5 \| \| 91 \| *S. saprophyticus* \| positive \| 0.25 \| 0.03 \| 0.5 \| 0.0019 \| 2 \| \| 99 \| *S. warneri* \| positive \| 0.03 \| 0.03 \| 1 \| 0.0019 \| 2 \| \| 94 \| *S. warneri* \| positive \| 0.25 \| 0.06 \| 1 \| 0.0019 \| 1 \| \| 95 \| *S. warneri* \| positive \| 0.25 \| 0.0019 \| 1 \| 0.0019 \| 2 \| \| 96 \| *S. warneri* \| positive \| 1 \| 0.003 \| 1 \| 0.0019 \| 2 \|   Where the MIC is expressed as >1024 ug/ml then resistance to all tested concentrations occurred. |  |
|  |  |
